# Awareness of Emotional Expressions in Cannabis Users: An Event-Related Potential Study

**DOI:** 10.3389/fpsyg.2019.00069

**Published:** 2019-02-01

**Authors:** Robert D. Torrence, Donald C. Rojas, Lucy J. Troup

**Affiliations:** ^1^Department of Pharmacy Practice, Wayne State University, Detroit, MI, United States; ^2^Department of Psychology, Colorado State University, Fort Collins, CO, United States; ^3^Strategic Hub for Psychology, Social Work, Health Behaviours and Addictions, University of the West of Scotland, Paisley, Scotland

**Keywords:** cannabis, ERP, emotion, facial expression, awareness

## Abstract

Cannabis use has been associated with anxiogenic effects when used in low frequency for a short duration, but cannabis can also have anxiogenic effects when used heavily for a long duration. Animal studies have indicated the neurobiological mechanisms related to cannabis and anxiety; however, research has been limited on the related neurocognitive mechanisms. Previous research has indicated that cannabis use is associated with alterations in event-related potentials (ERPs). The purpose of the current study was to examine anxiety related attentional processing of emotional expressions using ERP methods. We used a backward masking paradigm to restrict awareness of facial expressions (i.e., fearful, happy, and neutral). The results indicated that cannabis use was associated with differences in emotional processing. Specifically, the results suggested cannabis users had increased P1 amplitudes toward happy facial expressions compared to fearful and neutral. Additionally, cannabis users seemed to have reduced N170 hemisphere lateralization.

## Introduction

There has been a significant increase in cannabis use among adolescents between 2002 and 2015 (Substance Abuse and Mental Health Services Administration, 2016). Given that cannabis use has increased in availability and use, likely because of the increased legalization in the United States (National Conference of State Legislatures, 2018), it is important to understand the effects cannabis use has on the brain and behavior. Previous research has suggested that cannabis use was correlated with decreased memory, attention, and emotional processing (Broyd et al., 2016; Troup et al., 2016b, 2017; Lovell et al., 2018). Neuroanatomical differences have also been found between cannabis users and non-users: specifically in the amygdala, prefrontal cortex (PFC), and insula (Lorenzetti et al., 2016). The endocannabinoid system has been a target for treatment of anxiety related disorders (Rabinak and Phan, 2014; Korem et al., 2016); however, how cannabis might affect emotion processing is unclear. The main phytocannabinoid found in cannabis, Δ9-tetrahydrocannabinol (THC), has been suggested to have anxiolytic effects (Berrendero and Maldonado, 2002; Viveros et al., 2005; Patel and Hillard, 2006; Rubino et al., 2007), although other research has indicated that excessive cannabis use has anxiogenic effects (Viveros et al., 2005). Individuals with anxiety tend to have enhanced attention and processing of threat-related (Bar-Haim et al., 2007) and positive (Morel et al., 2014) stimuli. These differences in attentional processing can be measured using event-related potentials (ERPs) (Harrewijn et al., 2017). The aim of this study was to examine the residual effects of cannabis use on attention to emotional facial expressions when awareness was restricted, versus when awareness was not restricted using a backward masking paradigm.

Backward masking occurs when a target face is displayed for a short duration and then is immediately replaced by a mask stimulus (neutral face or scrambled face). Pessoa et al. (2005) conducted a behavioral study in which they varied the target face (fearful, happy, or neutral face) and target duration (17, 33, and 83 ms) to test the awareness threshold duration for the target face. The researchers found that a target face duration of 17 ms was below the awareness threshold for most of the participants (nine out of 11); at 33 ms, seven out of 11 participants scored above chance level in detecting the target face, and all of the participants were aware of the target face displayed for 83 ms. These results suggested there are individual differences in perceptual awareness and established that backward masking is most effective when the target faces are displayed for 17 ms or less.

Backward masking fMRI studies suggested the amygdala was more active for negative facial expressions compared with neutral or happy faces, even when awareness was restricted (Morris et al., 1998; Whalen et al., 1998; Suslow et al., 2006). Similarly, dot-probe task fMRI research found that the visual cortex had increased activity when attending toward fearful faces (Pourtois et al., 2006; Carlson et al., 2011), and visual cortex activity was correlated with amygdala activity (Carlson et al., 2009). Researchers have used ERPs to measure the time-course of processing facial expressions. A number of studies indicated that even when awareness of emotional facial expressions was restricted using backward masking, multiple ERP components were modulated by negative target faces (fear and anger) compared to non-negative faces (happy and neutral) (Pegna et al., 2008, 2011; Del Zotto and Pegna, 2015; Vukusic et al., 2017). The P1 ERP component has a positive peak around 80–120 ms in the lateral occipital electrodes. Participants with high trait anxiety had more enhanced P1 amplitude to happy faces compared to neutral, and there was no difference between fear and neutral (Morel et al., 2014); whereas other research suggested that P1 was more enhanced in high anxiety toward negative stimuli (Helfinstein et al., 2008; Holmes et al., 2008; Mueller et al., 2009; Harrewijn et al., 2017; Torrence and Troup, 2017). Overall the P1 component is thought to reflect an initial increase of attention in the extrastriate cortex (Mangun et al., 1997; Pourtois et al., 2005) and is more pronounced in anxiety. The N170 component is a negative deflection in the ERP waveform which peaks around 170 ms after stimulus onset and is found in lateral posterior electrodes, which typically has a right hemisphere lateralization (Bentin et al., 1996). A meta-analysis indicated that the N170 is sensitive to facial expression: particularly to anger, fear, and happiness expressions (Hinojosa et al., 2015). In addition, these studies found that the overall amplitude of N170 was more negative for faces displayed for a long duration compared to a short duration. That is, when awareness of a fearful face was restricted, the N170 was enhanced compared to neutral. The same was found when awareness was not restricted, but the amplitude in the aware condition was more negative overall. Source localization of the N170 was found to originate in the right extrastriate visual cortex (Pegna et al., 2008). However, another study indicated that emotional expression did not influence the N170 (Kiss and Eimer, 2008).

In addition to P1 and N170, the N2 component has also been suggested to indicate orientation to salient facial expressions regardless of awareness (Liddell et al., 2004; Vukusic et al., 2017). The N2 ERP component is the second negative peak occurring 180–300 ms and can be found in central electrodes (i.e., FZ, CZ, and PZ). Contrary to Liddell et al. (2004) and Pegna et al. (2008), Vukusic et al. (2017) only found N2 differences in unmasked conditions as opposed to masked. Other research suggested the N2 component involves cognitive control, or controlling actions (Folstein and Van Petten, 2008). Although the source of anterior N2 is debated, it is thought to originate in the medial frontal cortex (e.g., ACC) and the right inferior frontal cortex (Ridderinkhof and Ullsperger, 2004; Folstein and Van Petten, 2008; Aron et al., 2016). Lastly, the P3 ERP component has multiple subcomponents, but this article focuses on the later P3 between 400 and 600 ms found in central, posterior electrodes (Kiss and Eimer, 2008). The enhanced P3 amplitude reflects higher level emotional and attentional processing (Johnston et al., 1986; Polich, 2007). Previous research suggested that cannabis use modulates the P3 amplitude toward emotional facial expressions, particularly in implicitly processed (Troup et al., 2016b, 2017).

The main purpose of this study was to examine the residual effects of cannabis use on processing facial expression under restricted awareness. Given the relationship between awareness and processing of emotional expressions and anxiety, as well as the effects cannabis has on anxiety, we hypothesized that individuals that use cannabis would have residual attenuation in their ERPs in responses to emotional faces (similar to what researchers have found in low anxiety). More specifically, cannabis users would have reduced (less enhanced) P1, N170, N2, and P3 amplitudes to fearful and happy facial expression. These results would indicate that processing of emotional expressions could be a neutral cognitive mechanism of the anxiolytic effects of cannabis use.

## Materials and Methods

### Participants

Forty adults from Colorado State University and members of the Fort Collins community participated in this study (23 females, 1 non-binary). A predetermined age range was set from 18 to 35 years old (*M* = 23.75, *SD* = 3.94; range = 19–35). Participants were recruited from students enrolled in summer courses and received extra credit. The students also received extra credit for each person they recruited from the community. Thirty-three reported they were right handed and four indicated they were ambidextrous. Only one participant reported consuming alcohol within the last 24 h (but not 8 h prior to the study); regular alcohol use was not assessed. None of the participants reported use of prescription or illicit drugs. The participants reported normal or corrected vision and no history of brain injury or psychiatric disorders. All participants provided written informed consent before participating. This experiment was approved by the Colorado State University Institutional Review Board.

### Questionnaires

A custom personal inventory was used to assess recent use of caffeine, tobacco, cannabis, and alcohol, as well as age, vision, history of disorders, and medicines used. To divide the participants into cannabis users and non-users, the Recreational Cannabis Use Questionnaire (RCUE; Troup et al., 2016b) was used. The RCUE asked the participants about their history of cannabis use, including average monthly use and preferred method (e.g., smoking, edibles, dabs, etc). Participants were instructed to count “use” as a single time they consumed (any amount) to feel high. Cannabis users were defined as using more than monthly for at least 1 year. Non-users were defined as never using or not using in the last 2 years. The Center for Epidemiological Studies Depression scale (CESD; Radloff, 1977), the state portion of the State-Trait Anxiety Inventory (STAI; Spielberger et al., 1983), and PTSD Checklist for DSM-5 (PCL-5; Weathers et al., 2013) were collected for exploratory analyses.

### Awareness Task

**FIGURE 1:**
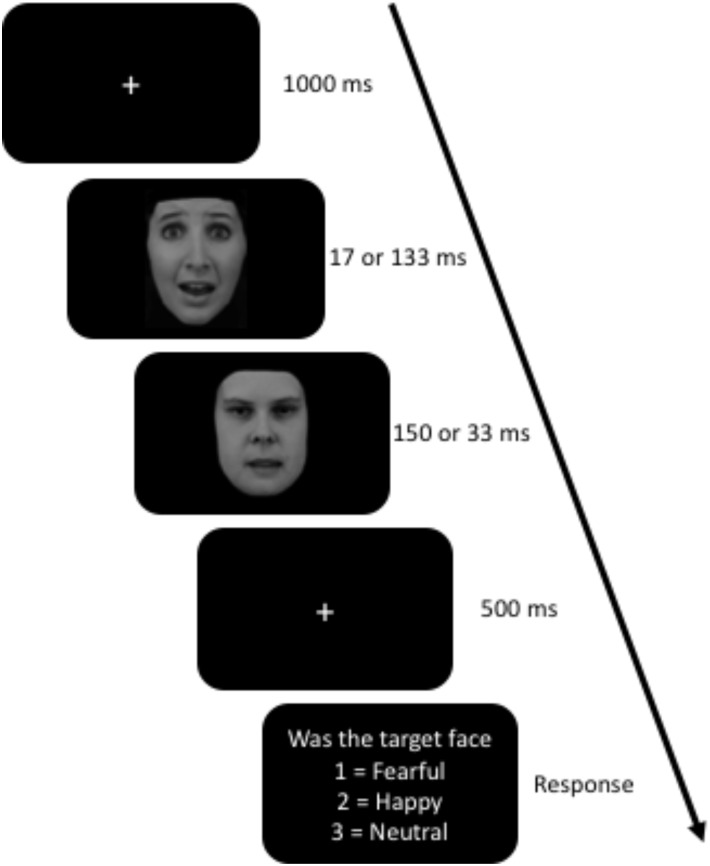
Awareness Task. The target face was the first face to appear for either 17 or 133 ms and was either a fearful, happy, or neutral face. The masking face, the second one, was always a neutral face with open mouth. Facial expressions (AF13AFS and AF16NES) obtained from The Karolinska Directed Emotional Faces database (Lundqvist et al., 1998).

The facial stimuli were neutral, happy, and fearful facial expressions from the Karolinska Directed Emotional Faces (KDEF) database (Lundqvist et al., 1998). All non-face stimuli (i.e., background, hair, neck and ears) were cropped and the faces were grayscale. The following were the KDEF ids for fear, happy, and neutral: AF13, AF14, AF19, AM09, and AM1022. The task was programmed and displayed using Stim2 software (Compumedics USA, Inc., Charlotte, NC, United States). This task displayed one face at a time in the center of the screen at 3° × 4.4° of the visual angle on a 20-inch, 144 Hz LCD monitor. Trials started with a white fixation cue (++) for 1000 ms on a black screen, followed by the target face (fearful, happy, or neutral expression) and immediately replaced with the masking face (neutral face with open mouth). The following were the KDEF ids for the target faces: AF13, AF14, AF19, AM09, and AM1022. The masking faces ids were AF16NES and AM03NES. In the restricted awareness (masked) condition, the target face was programmed to display for 16.66 ms followed by 150 ms mask. The aware condition (unmasked) was programmed to display the target face for 133.33 ms and the mask was displayed for 33.33 ms. In both conditions, there was a stimulus present for the same amount of time (166.66 ms). However, after all the data was collected, we tested the actual stimulus duration using a photodiode (AMS Technologies, model TSL257) and Arduino Micro microcontroller. We found that the 16.66 ms duration was actually 30 ms, 150 ms was 151 ms, 133 ms was 135 ms, and 33 ms was 44 ms. After the masking face, a fixation cue was present for 500 ms followed by a question. The question asked the participants whether the target face was fearful, happy, or neutral, to which the participants responded using the number pad on a keyboard (1, 2, or 3) (see Figure 1). The participants were told before the task to use their gut instinct and respond as quickly as possible. The task was divided into 13 blocks with 72 trials in each block for a total of 936 trials. Within each block, there were equal number of trial types (duration and emotion) presented randomly. The task took between 35 and 45 min, depending on how long the participant took to respond, and how long they took between blocks.

### EEG Data Collection

The acquisition software used to collect the EEG data was Curry 7 using 33 Ag/AgCl electrodes from a SynAmpsRT 64-channel QuickCap (Compumedics USA, Inc., Charlotte, NC, United States). The following electrodes were used for recording: FP1, FP2, F7, F3, FZ, F4, F8, FC5, FC1, FC2, FC6, T7, C3, CZ, C4, T8, CP5, CP1, CP2, CP6, P7, P3, PZ, P4, P8, PO7, PO3, POZ, PO4, PO8, O1, and O2 with the right mastoid as a reference during acquisition, and a ground electrode located between FCZ and FZ. Neurocompumedics Quick Gel was used to reduce impedances which were kept below 10 kΩ. To measure eye movements and blinks we used horizontal electro-oculogram (HEO) electrodes placed on the outer canthi of the left and right eye. The EEG sampling rate was set to 500 Hz and the recording bandwidth was DC to 250 Hz.

The raw data was preprocessed using EEGLAB and ERPLAB (Delorme and Makeig, 2004; Lopez-Calderon and Luck, 2014). We first referenced the data to a common average reference and then applied a Butterworth bandpass filter of 0.1–40 Hz with a roll-off slope of 12 dB/octave. The data were then epoched from -200 to 1000 ms around the onset of the masked facial expression. Trials were rejected for artifacts using a simple voltage threshold (-100 to 100 μV) and by visual inspection. Participants with more than 30% of trials rejected were excluded from the study. Mean amplitudes were calculated for each ERP component time locked to target face onset for all trials (correct and incorrect trials). Previous research was used to determine the ERP components’ time window and electrode location. P1 (80 – 120 ms) was taken from the O2 electrode (Suway et al., 2013), N170 (150 – 190 ms) was taken from P7 and P8 electrodes (Carlson and Reinke, 2010), N2 (180 – 300 ms) was examined using FZ, CZ, and PZ (Vukusic et al., 2017), and P3 (400 – 600 ms) from PZ electrode (Kiss and Eimer, 2008).

### Data Analysis

We used uncorrected *t*-tests to examine group differences in questionnaires and age. The behavioral data was calculated as a percent correct for each emotion in each duration (masked or unmasked). A 3 (emotion) × 2 (duration) × 2 (group) ANOVA was used for the behavioral data, P1, and P3. For N170 we used a 3 (emotion) × 2 (duration) × 2 (hemisphere) × 2 (group) ANOVA with P7 and P8 electrodes for hemisphere. N2 was examined using a 3 (emotion) × 2 (duration) × 3 (electrode) × 2 (group) ANOVA. Greenhouse–Geisser and Bonferroni corrected comparisons were used when appropriate.

## Results

Four participants were removed from the study: one had 73% of their trials rejected, two reported vision problems (one had an under developed left optic nerve and the other had nystagmus), and the fourth was stopped early due to all the electrodes going over the impendence threshold. This left 18 cannabis users (10 females) and 18 non-users (11 females, 1 non-binary). The average monthly use for the cannabis users was 27.33 (*SD* = 31.08) with a range of 1–120 times a month. The participant that reported one instance of use in the last month indicated that he used almost daily previously and he was cutting back on their use. One other participant used at least weekly, nine multiple times a week, and seven used more than daily. Years of use ranged from 2 to 19 years. The youngest a participant first used cannabis was 12 and the oldest was 19. The RCUE did ask about the number of grams used per month but many participants did not know how to answer and therefore left it blank. Additionally, there was wide variety of consumption methods (edibles, smoking methods, dabs, etc.) and type of cannabis (indica or sativa) between and within participants, which made using grams per month a less reliable measure. Two cannabis users did not complete the STAI and one non-user did not complete the CES-D, their data were excluded from any analyses involving those questionnaires. There were no significant differences between age, STAI, CES-D, or PCL-5 (Table 1). Previous research that examined group differences in a similar task used 14 and 12 participants (Vukusic et al., 2017). All relevant data has been reported in this manuscript.

**Table 1:** Mean and standard deviations of age, STAI, CES-D, PCL-5, age of onset, and monthly use.

	Cannabis users		Non-users			
	(*n* = 18; 10 F)		(*n* = 18; 11 F, 1 NB)			
	*M*	*SD*	*M*	*SD*	*t*	*p*
Age	23.94	4.19	23.56	3.78	-0.29	0.77
STAI-State	37.13	11.62	33.83	8.48	0.17	0.35
CES-D	17.06	9.6	13.12	6.87	-1.37	0.18
PCL-5	16.83	19.59	14.28	14.12	-0.45	0.66
Age Onset	15.88	2.03	–	–		
Monthly use	27.33	31.08	–	–		


*There were no significant differences between groups in any of the measures. F, females; NB, non-binary; STAI-State, state portion of the State-Trait Anxiety Inventory; CES-D, Center for Epidemiological Studies Depression scale; PCL-5, PTSD Checklist for DSM-5*.

### Behavioral

**FIGURE 2:**
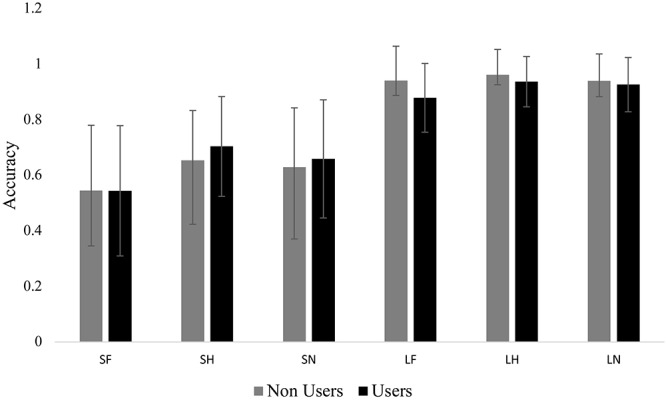
Mean accuracy for each group in each condition. Bar graph displaying the mean accuracy scores with standard deviation bars. The short condition (i.e., masked) was significantly lower than the long condition (i.e., unmasked). There were no group differences and no differences within long and short. SF, short fear; SH, short happy; SN, short neutral; LF, long fear; LH, long happy; and LN, long neutral.

We found a significant main effect for emotion *F*(1.84,62.52) = 4.42, *p* = 0.018, $\eta_{p}^{2}$ = 0.115. Bonferroni *post hoc* comparisons indicated that participants had less accuracy to fearful (*M* = 0.73, *SE* = 0.02) compared to happy (*M* = 0.81, *SE* = 0.02). There was also a significant main effect for duration *F*(1,34) = 401.70, *p* < 0.001, $\eta_{p}^{2}$ = 0.922. Subjects were less accurate in the masked condition (*M* = 0.62, *SE* = 0.02) compared to unmasked condition (*M* = 0.93, *SE* = 0.01). The interaction between group and duration approached significance, *F*(1,34) = 3.75, *p* = 0.061, $\eta_{p}^{2}$ = 0.099. While there were no differences between groups, both groups had within group differences between masked and unmasked. Even though there were no group effects, there was variability in accuracy especially in the masked condition (Figure 2).

### P1

We found a significant main effect for emotion, *F*(1.43,48.64) = 8.87, *p* = 0.002, $\eta_{p}^{2}$ = 0.207. Happy (*M* = 4.36, *SE* = 0.49) was significantly greater than fearful (*M* = 3.93, *SE* = 0.48, *p* = 0.004) and neutral (*M* = 3.95, *SE* = 0.47, *p* = 0.018). No difference was found between fear and neutral. There was also a significant main effect for duration, *F*(1,34) = 14.88, *p* < 0.001, $\eta_{p}^{2}$ = 0.304. The masked condition (*M* = 4.29, *SE* = 0.47) was significantly greater than unmasked (*M* = 3.87, *SE* = 0.49, *p* < 0.001).

**FIGURE 3:**
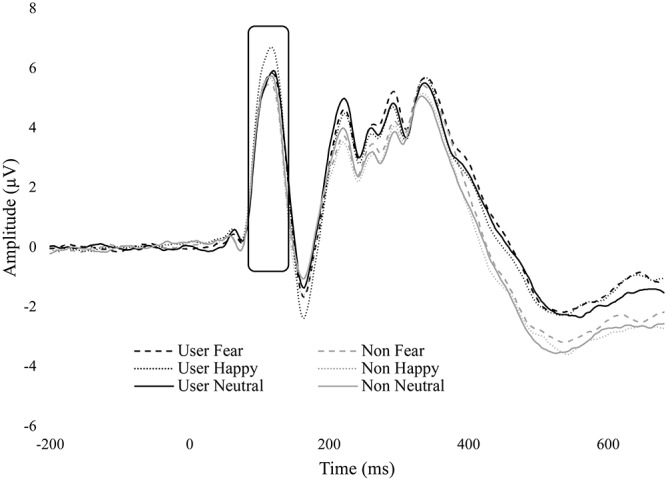
ERP wave from O2. ERP waveform from electrode O2 displaying the P1 component. Cannabis users had greater amplitude for happy.

No significant group interactions were observed. However, there was a trend in emotion by group, *F*(1.43,48.64) = 3.03, *p* = 0.074, $\eta_{p}^{2}$ = 0.081. There were no differences between emotions within non-users, but within cannabis users, happy (*M* = 4.58, *SE* = 0.70) was greater than fear (*M* = 3.99, *SE* = 0.68, *p* = 0.005) and neutral (*M* = 3.90, *SE* = 0.67, *p* = 0.004) (Figure 3).

### N170

There was a significant main effect for emotion, *F*(2,68) = 24.31, *p* < 0.001, $\eta_{p}^{2}$ = 0.417. All three emotions were significantly different from each other. Happy (*M* = -3.19, *SE* = 0.45) was more enhanced than fearful (*M* = -2.89, *SE* = 0.45), and both were more enhanced than neutral (*M* = -2.50, *SE* = 0.41), *p*s < 0.002. There was also a significant main effect for duration, *F*(1,34) = 10.90, *p* = 0.002, $\eta_{p}^{2}$ = 0.423. The unmasked faces (*M* = -2.99, *SE* = 0.44) elicited an enhanced N170 compared to masked (*M* = -2.73, *SE* = 0.43), *p* = 0.002. Hemisphere also had a significant main effect, *F*(1,34) = 7.47, *p* = 0.010, $\eta_{p}^{2}$ = 0.180. Overall the P8 electrode (*M* = -3.56, *SE* = 0.56) was more negative than P7 (*M* = -2.16, *SE* = 0.44), *p* = 0.010. No other main effects or interactions were significant.

**FIGURE 4:**
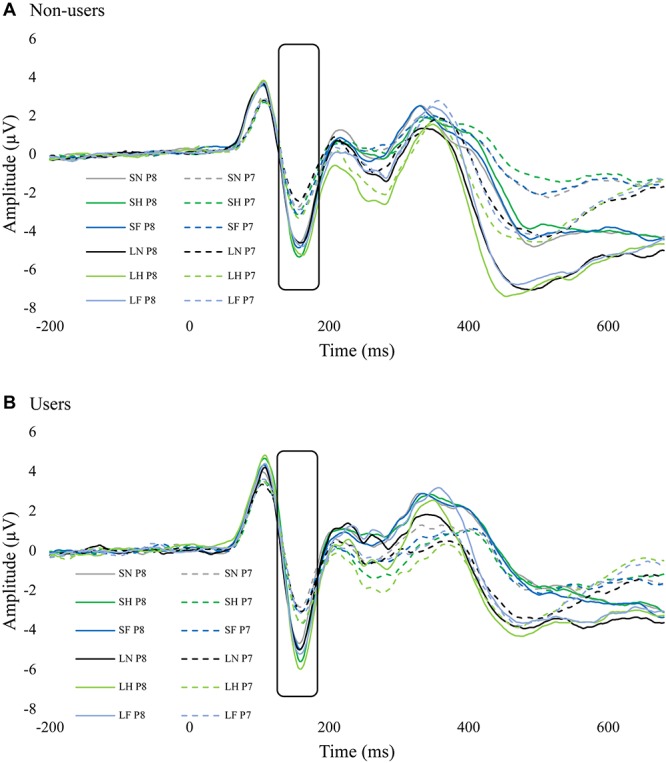
ERP waves from P7 and P8 for each group. These two figures display the ERP waveforms from electrodes P7 and P8 for non-users **(A)** and cannabis users **(B)**. There was significant hemisphere lateralization in non-users but not in users. SF, short fear; SH, short happy; SN, short neutral; LF, long fear; LH, long happy; and LN, long neutral.

The interaction between group, emotion, duration, and hemisphere was not significant, *F*(2,68) = 2.76, *p* = 0.071, $\eta_{p}^{2}$ = 0.075. An exploratory Bonferroni *post hoc* comparisons indicated that there was hemisphere lateralization (i.e., enhanced N170 in P8 compared to P7) in non-users for masked happy (*p* = 0.017), unmasked happy (*p* = 0.034), masked neutral (=0.038), and unmasked neutral (*p* = 0.015). Cannabis users, however, did not have any significant differences in hemisphere lateralization (Figure 4 and Table 2).

**Table 2:** N170 overall mean and standard error for emotion.

	Non-users					
	Masked			Unmasked		
	P7	P8	*p*	P7	P8	*p*
Happy	-1.94 (0.63)	-3.85 (0.83)	0.017	-2.34 (0.68)	-4.12 (0.84)	0.034
Neutral	-1.72 (0.59)	-3.22 (0.76)	0.038	-1.53 (0.63)	-3.39 (0.76)	0.015
Fearful	-2.14 (0.64)	-3.47 (0.82)	0.071	-2.18 (0.64)	-3.68 (0.83)	0.062

	**Cannabis users**					
	**Masked**			**Unmasked**		
	**P7**	**P8**	***p***	**P7**	**P8**	***p***

Happy	-2.68 (0.63)	-3.63 (0.83)	0.168	-2.75 (0.68)	-4.24 (0.83)	0.150
Neutral	-1.84 (0.59)	-2.82 (0.76)	0.222	-2.21 (0.63)	-3.28 (0.76)	0.074
Fearful	-2.15 (0.64)	-3.29 (0.82)	0.121	-2.44 (0.64)	-3.73 (0.83)	0.109


*P-values were Bonferroni corrected*.

### N2

There was a significant main effect for emotion, *F*(2, 68) = 15.59, *p* < 0.001, $\eta_{p}^{2}$ = 0.314. The N2 amplitudes for fear (*M* = -0.67, *SE* = 0.28) and neutral (*M* = -0.62, *SE* = 0.26) were more negative than happy (*M* = -0.39, *SE* = 0.28, *p*s < 0.001), there was no difference between fear and neutral. There was also a significant main effect for electrode, *F*(1.12,37.68) = 35.20, *p* < 0.001, $\eta_{p}^{2}$ = 0.509. FZ (*M* = -2.30, *SE* = 0.47) was more negative than CZ (*M* = -1.15, *SE* = 0.36), which both were more negative than PZ (*M* = 1.78, *SE* = 0.35, *p*s < 0.001). There was a significant interaction between emotion and duration, *F*(2,68) = 2.60, *p* = 0.002, $\eta_{p}^{2}$ = 0.167. *Post hoc* comparisons revealed no differences within the masked condition but in the unmasked condition, happy (*M* = -0.22, *SE* = 0.30) was greater than fear (*M* = -0.65, *SE* = 0.30, *p* < 0.001) and neutral (*M* = -0.61, *SE* = 0.28, *p* < 0.001), no difference between fear and neutral. The interaction between emotion, duration, and electrode was also significant, *F*(2.53,85.98) = 9.49, *p* < 0.001, $\eta_{p}^{2}$ = 0.218. Within FZ, there were no differences between masked emotional expression; however, in the unmasked condition, happy (*M* = -1.54, *SE* = 0.56) was greater than fear (*M* = -2.52, *SE* = 0.49, *p* < 0.001) and neutral (*M* = -2.47, *SE* = 0.49, *p* < 0.001). Similarly, in CZ, no differences in masked faces but in unmasked, happy (*M* = -0.88, *SE* = 0.40) was greater than fear (*M* = -1.44, *SE* = 0.39, *p* < 0.001) and neutral (*M* = -1.29, *SE* = 0.35, *p* < 0.001). No differences were found within PZ.

**FIGURE 5:**
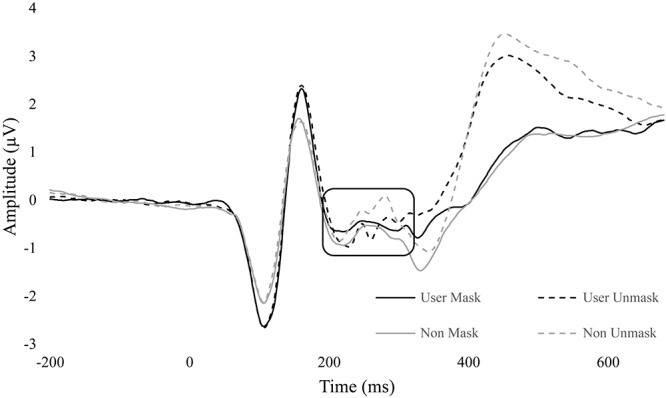
ERP wave from FZ, CZ, and PZ. ERP waveform from electrodes FZ, CZ, and PZ to display differences in N2 (180 – 300 ms). Non-user unmasked was significantly greater than non-user mask. No difference between conditions in users.

There was also a significant interaction between group and duration, *F*(1,34) = 4.66, *p* = 0.038, $\eta_{p}^{2}$ = 0.120. There were no between-group differences in duration. However, within non-users, masked faces (*M* = -0.72, *SE* = 0.37) had more negative amplitudes than unmasked (*M* = -0.35, *SE* = 0.40, *p* = 0.019). Within cannabis users, there was no difference between masked (*M* = -0.54, *SE* = 0.37) and unmasked (*M* = -0.63, *SE* = 0.41, *p* = 0.568) conditions (Figure 5).

### P3

**FIGURE 6:**
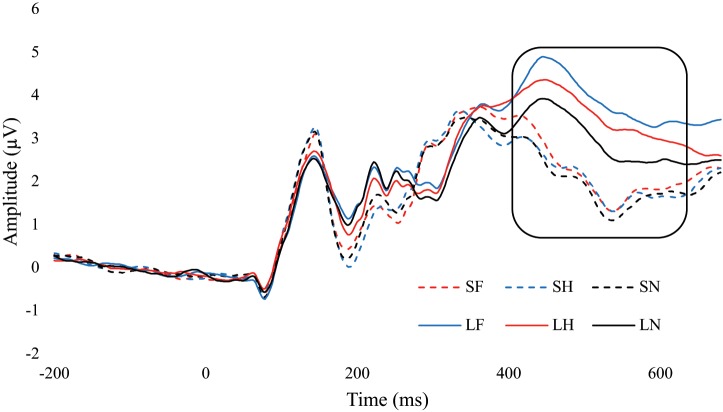
ERP wave from PZ. ERP waveform from the PZ electrode to examine the difference in the P3 component (400 – 600 ms). SF, short fear; SH, short happy; SN, short neutral; LF, long fear; LH, long happy; and LN, long neutral.

There was a significant main effect for emotion, *F*(2, 68) = 11.01, *p* < 0.001, $\eta_{p}^{2}$ = 0.245. Fear (*M* = 3.15, *SE* = 0.32) had significantly greater amplitude than neutral (*M* = 2.56, *SE* = 0.30, *p* < 0.001), and fear was approaching significance compared to happy (*M* = 2.91, *SE* = 0.33, *p* = 0.081). Happy was also approaching significance with neutral, *p* = 0.053. There was a significant main effect for duration, *F*(1,34) = 90.21, *p* < 0.001, $\eta_{p}^{2}$ = 0.726. Amplitudes were significantly greater for unmasked faces (*M* = 3.62, *SE* = 0.34) compared to masked (*M* = 2.13, *SE* = 0.30, *p* < 0.001). There was also an interaction between emotion and duration, *F*(2,68) = 7.61, *p* = 0.001, $\eta_{p}^{2}$ = 0.183. Within the masked condition there were no differences between fear, happy, and neutral, *p*s > 0.261. In the unmasked condition, fear (*p* < 0.001) and happy (*p* = 0.008) were greater than neutral. Differences in fear and happy were approaching significance, *p* = 0.056. Additionally, each emotional expression had greater amplitude in unmasked compared to masked, *p*s < 0.001 (Figure 6 and Table 3).

**Table 3:** P3 overall mean and standard error for emotion and duration.

	Masked	Unmasked	*p*
Happy	2.13 (0.30)	3.70 (0.38)	0.000
Neutral	1.99 (0.31)	3.13 (0.31)	0.000
Fearful	2.26 (0.31)	4.04 (0.37)	0.000


*No differences between emotion within masked. In unmasked, fear and happy were greater than neutral, *p*s < 0.008*.

No group interactions were found for P3 amplitude in emotion *F*(2, 68) = 0.07, *p* = 0.935, $\eta_{p}^{2}$ = 0.002, duration *F*(2,68) = 0.94, *p* = 0.339, $\eta_{p}^{2}$ = 0.027, and emotion by duration *F*(2,68) = 1.09, *p* = 0.304, $\eta_{p}^{2}$ = 0.030.

### Exploratory Analysis

An exploratory analysis was conducted to examine potential differences between males and females. There were no ERP differences between males and females. There were also no correlations between questionnaire data and ERP components.

## Discussion

The behavioral results suggested that masked facial expressions were not completely below the awareness threshold; each expression was greater than chance level (33.33%). Given that the actual refresh rate for the target face in the masked condition was 30 ms and not 16.66 ms, some of the participants might have been aware (Pessoa et al., 2005). However, on average, the participants were less accurate in correctly reporting the expression in the masked condition compared to unmasked. This suggests that awareness was restricted, but maybe not completely below the awareness threshold. Although, no difference in accuracy between cannabis users and non-users, suggesting that cannabis use did not affect subjective perceptual awareness. Since the behavioral data was insignificant, the ERP differences between groups are likely caused by differences in emotional processing, in general, rather than awareness of the emotional expressions (Troup et al., 2016b, 2017).

Cannabis users had increased amplitude toward happy facial expressions compared to fear and neutral expression. This result would suggest that cannabis users had increased early processing of happy facial expressions, independent of awareness. Previous research by Morel et al. (2014) indicated that individuals with high levels of trait anxiety showed a similar effect, in that there was enhanced P1 amplitude for happy faces but no difference in fear and neutral. In their experiment, the facial expressions (happy, fear, and neutral) were displayed for 500 ms and awareness was not restricted. The current results did not address whether cannabis users had greater anxiety. In fact, state anxiety, depression, and posttraumatic stress symptoms (PTSS) were statistically equal between users and non-users. However, our results suggest that cannabis users had increased attention toward a positive salient stimulus which is a similar finding to Morel et al. (2014) who also found an increased attentional bias toward positive stimuli in participants with high trait anxiety. Neither our results nor Morel et al. (2014) found differences between fear and neutral emotional expression which is dissimilar to other research (Helfinstein et al., 2008; Holmes et al., 2008; Mueller et al., 2009; Harrewijn et al., 2017; Torrence and Troup, 2017).

In previous backward masking studies, P1 differences were not modulated by emotional expression or masking conditions (Pegna et al., 2008, 2011; Del Zotto and Pegna, 2015). The results of the current study did suggest that across all participants, P1 was greater in the masked condition compared to unmasked with happy having a greater P1 amplitude than fear and neutral. This is the first study, however, to suggest that masked faces elicited a greater P1 amplitude than unmasked faces. The P1 component is thought to reflect increased processing in the amygdala and visual cortex (Pourtois et al., 2004; Vuilleumier and Pourtois, 2007; Carlson et al., 2009). Etkin et al. (2004) found that masked faces increased basolateral amygdala activity and unmasked increased dorsal amygdala. They proposed that the visual, cingulate, and prefrontal connections of the basolateral amygdala represent the neural system related to the enhanced processing of masked faces. This could be a possible explanation of why we found enhanced P1 amplitudes in the masked condition.

The N170 results suggested that regardless of masking condition, emotional facial expressions had more enhanced N170 amplitudes. Specifically, happy was more enhanced than fear, and both were more enhanced than neutral. This is consistant with a recent review that suggested N170 is modulated by emotional expressions (Hinojosa et al., 2015). Additionally, we found that N170 for unmasked faces was more enhanced than masked faces. Del Zotto and Pegna (2015) found a similar result, that unmasked faces elicited a more negative N170 than masked. However, they also found an interaction between expression and duration within the masked condition, and that fearful faces had more enhanced amplitudes than neutral, which we did not find in the current study. We did observe an interesting group interaction in N170: within non-users, each emotion within each masking condition showed hemisphere lateralization, whereas cannabis users did not have this effect. A similar effect was found by Vukusic et al. (2017) in participants with high autistic traits, which might indicate that faces are not as salient to cannabis users as they are to non-users. Alternatively, Maurer et al. (2008) examined N170 lateralization for faces and words, and found reduced hemisphere lateralization for faces when the faces were presented one after another within the same block as compared to when faces and words were alternated. Their results suggested that a reduction in hemisphere lateralization indicated habituation in face processing. Taken together, cannabis users may have increased habituation to facial expressions compared to non-users.

This study only found N2 differences within the unmasked condition, similar to Pegna et al. (2008). However, Pegna et al. (2008) found differences in unmasked fear and neutral while we only found differences in happy compared to fear and neutral. Additionally, our results contradicted Liddell et al. (2004) and Vukusic et al. (2017) in that we found no emotional expression differences within the masked condition. Our results also suggested that N2 was more prominent in frontal and central electrodes as opposed to posterior and may be related more with cognitive control (Folstein and Van Petten, 2008), whereas a more posterior N2 might be related to orientation of attention (Luck and Hillyard, 1994a,b; Dowdall et al., 2012; Tan and Wyble, 2015; Diao et al., 2017). Group comparisons within non-users revealed an enhanced N2 for unmasked compared to masked facial expression. This difference, however, was not seen within cannabis users suggesting that non-users had better cognitive control over their response to unmasked facial expressions than cannabis users.

Although there were overall differences in P3 amplitudes between facial expression, differences were only observed within the unmasked condition. Specfically, fear elicited a greater P3 than happy, which was greater than neutral. Liddell et al. (2004) and Kiss and Eimer (2008) found similar effects and suggested that P3 amplitudes were related to higher emotional processing which requires percpetual awareness. However, unlike Troup et al. (2016b), these results suggested cannabis use was not related to differences in emotional processing as measured by P3. One explanation could be that Troup et al. (2016b) found differenes in implicit emotional expression processing and not in explicit. Given the nature of the current task, the participants were asked to pay attention to the target face expression. Therefore, when cannabis users are asked to pay attention to the emotion, there are no differences between them and non-users (Troup et al., 2016b).

There were limitations to this study. Firstly, the behavioral data suggests that there was variablitiy in awareness to facial expression (Figure 1). Although we found no differences between groups in STAI, CES-D, and PCL-5, we did not control for high and low levels of anxeity, depression, and PTSD. Given that some individuals self medicate with cannabis for these disorders (Crippa et al., 2009; Troup et al., 2016a), it would be interesting to see the effects cannabis has on participants with high levels of anxiety. Additionally, we did not control for type of cannabis use. Since cannabis is recreationally legal in the State of Colorado, users have a wide variety of strains (e.g., indica and sativa) and method of use (e.g., flower, concentrates, edidbles, etc). Given that different ratios of cannabinoids affect the brain differently (Schubart et al., 2011), it would be worth exploring the effects on face processing. Early face processing requires the amygdala (Morris et al., 1996, 2001; Whalen et al., 1998; Anderson and Phelps, 2001; Vuilleumier et al., 2004; Suslow et al., 2006; Bach et al., 2015) and cannabis affects amygdala activity (Phan et al., 2008; Rabinak et al., 2014; Rabinak and Phan, 2014). The ERP methods used in this current study cannot directly measure amygdala activity even though the amygdala likely influences the ERP results. Future research could use fMRI to explore amygdala activity in cannabis users using a similar backward masking paradigm.

This study was the first to examine the effects of residual cannabis use on perceptual awareness of emotional facial expressions as measured by ERPs. We found that overall, cannabis use did not effect perceptual awareness but was related to differences in emotional processing. The results largely support findings from previous research (Liddell et al., 2004; Kiss and Eimer, 2008; Del Zotto and Pegna, 2015; Vukusic et al., 2017) that discribed how the brain responds to faces below and above the awareness threshold. Additionally, we observed differences in facial processing between cannabis users and non-users. Specifically, cannabis users displayed a hypervigilance toward happy faces (regardless of awareness), facial habituation, and reduced cognitive control to unmasked faces.

## Ethics Statement

All research wasconducted according to Colorado State Universities Internal Review Board and adheres to strict ethical guidelines on conducting research with human participants outlined by Colorado State University and Federal Laws. Protocol Number 12-3716H.

## Author Contributions

All authors have had an active role in preparing the manuscript. RT collected and analyzed the data under the supervision of his doctoral advisor LT and co-advisor DR. Inception and design was primarily RT and LT. DR was instrumental in data analysis and methods. All authors contributed to the writing of the manuscript which was prepared by RT.

## Conflict of Interest Statement

The authors declare that the research was conducted in the absence of any commercial or financial relationships that could be construed as a potential conflict of interest.
